# Increased levels of oral *Streptococcus*-derived d-alanine in patients with chronic kidney disease and diabetes mellitus

**DOI:** 10.1038/s41598-022-26175-1

**Published:** 2022-12-16

**Authors:** Yusuke Nakade, Yasunori Iwata, Norihiko Sakai, Masashi Mita, Maiko Nakane, Kenji Hamase, Wataru Suda, Tadashi Toyama, Shinji Kitajima, Akinori Hara, Miho Shimizu, Chikako Ogushi, Kengo Furuichi, Yoshitaka Koshino, Hidetoshi Morita, Masahira Hattori, Takashi Wada

**Affiliations:** 1 Department of Nephrology and Laboratory Medicine, Kanazawa University, 13-1 Takara-machi, Kanazawa, 920-8641 Japan; 2 Department of Clinical Laboratory, Kanazawa University, 13-1 Takara-machi, Kanazawa, Japan; 3 Division of Infection Control, Kanazawa University, 13-1 Takara-machi, Kanazawa, Japan; 4 Division of Blood Purification, Kanazawa University, 13-1 Takara-machi, Kanazawa, Japan; 5 KAGAMI Co., Ltd., 7-18 Saitobaiohiruzu Center 308, Ibaragi, Osaka Japan; 6 Graduate School of Pharmaceutical Sciences, Kyushu University, 3-1-1 Maidashi, Higashi-ku, Fukuoka, Japan; 7 RIKEN Center for Integrative Medical Sciences, 1-7-22 Suehiro-cho, Tsurumi-ku, Yokohama, Kanagawa Japan; 8 Graduate School of Frontier Sciences, The University of Tokyo, 5-1-5 Kashiwanoha, Kashiwa, Chiba Japan; 9 Department of Nephrology, Kanazawa Medical University, 1-1 Uchinada, Kahoku, Ishikawa Japan; 10 Department of Internal Medicine, Mizuho Hospital, 422-1 Tsubata, Kahoku, Ishikawa Japan; 11 Graduate School of Environmental and Life Science, Okayama University, 1-1-1 Tsushima-naka, Okayama, Japan

**Keywords:** Kidney diseases, Microbiome, Diagnostic markers

## Abstract

The number of patients on hemodialysis is increasing globally; diabetes mellitus (DM) complications is the major cause of hemodialysis in patients with chronic kidney disease (CKD). The d-amino acid (AA) profile is altered in patients with CKD; however, it has not been studied in patients with CKD and DM. Furthermore, bacteria responsible for altering the D-AA profile are not well understood. Therefore, we examined the D-AA profiles and associated bacteria in patients with CKD, with and without DM. We enrolled 12 healthy controls and 54 patients with CKD, with and without DM, and determined their salivary, stool, plasma, and urine chiral AA levels using two-dimensional high-performance liquid chromatography. We performed 16S rRNA gene sequencing analysis of the oral and gut microbiota to determine the association between the abundance of bacterial species and D-AA levels. Plasma d-alanine and d-serine levels were higher in patients with CKD than in healthy adults (*p* < 0.01), and plasma d-alanine levels were higher in patients with CKD and DM than in those without DM. The abundance of salivary *Streptococcus*, which produced d-alanine, increased in patients with CKD and DM and was positively correlated with plasma d-alanine levels. Patients with CKD and DM had unique oral microbiota and d-alanine profiles. Plasma d-alanine is a potential biomarker for patients with CKD and DM.

## Introduction

The number of patients on hemodialysis (HD) is increasing^1^, with diabetes mellitus (DM) being the major cause in patients with chronic kidney disease (CKD)^2,3^. Hence, identifying biomarkers and treatments for diabetic nephropathy is a global medical need of high priority. Diabetes onset causes hyperglycemia and glomerular hyperfiltration, which leads to nephron hypertrophy and proteinuria. In addition, podocytes become enlarged to accommodate the increased filtration surface. These conditions cause glomerular sclerosis and loss of nephrons, leading to the progression of CKD. In contrast, the cause of CKD in patients without DM is a paucity of nephrons per body mass, due to a poor nephron endowment from birth, obesity, pregnancy, renal aging, or injury-related nephron loss.

The majority of patients with CKD and DM are older adults with metabolic syndrome. Therefore, they often have age- and injury-related CKD prior to the onset of DM^4–7^, which affects the prognosis of the kidney and is not adequately described by the term “diabetic nephropathy”. Kidney biopsy studies have demonstrated a variety of nondiabetic kidney damage in patients with diabetic nephropathy^8^; therefore, the concept of “CKD with DM” has been proposed in addition to the terms “diabetic nephropathy” and “diabetic kidney disease”^9^. In the present study, we focused on the pathophysiology of patients with kidney disease complicated by DM.

Advances in analytical technology have enabled the separation of amino acids (AAs) into d- and l-optical isomers. The d-form was believed to be rare in the human body. However, the localization and function of d-isomers have been clarified with the advent of novel technologies^10–13^. Furthermore, it has become clear that bacteria are the main source of D-AAs^10,11,14^, and the levels of D-AAs reflect changes in the microbiota in various pathological conditions. We have previously reported that acute kidney injury (AKI) alters the gut microbiota and the levels of its metabolites d-serine (D-Ser)^10^ and d-alanine (D-Ala)^11^. In addition to patients with AKI, those with CKD^15,16^ and DM^17^ also exhibit changes in the gut microbiota. Altered blood D-AA profiles have consistently been reported in patients with CKD^13^. However, D-AA profiles have not been studied in patients with CKD and DM. Furthermore, bacteria responsible for the alteration of the D-AA profile remain unknown. Therefore, we examined the D-AA profiles and associated bacteria in patients with CKD, with and without DM.

## Methods

### Study population and sample collection

This prospective observational study included 66 participants (Table 1). Healthy adults, who had no infections, cancer, fever, diarrhea, or kidney disease (estimated glomerular filtration rate [eGFR] > 60 mL/min/1.73 m^2^), were enrolled in the control group. The following subsets of participants were enrolled in the kidney disease groups: patients with CKD (with and without DM) and patients on HD (with and without DM). CKD was defined as an eGFR of ≤ 60 mL/min/1.73 m^2^. Patients who were treated with immunosuppressive drugs and antibiotics were excluded, as were those with suspected infectious diseases, a temperature > 37 °C, diarrhea, and cancer. Saliva, stool, blood, and urine samples were simultaneously collected from each participant between 2013 and 2019 at the Kanazawa University Hospital and Mizuho Hospital.

**Table 1:** Clinical characteristics of the study participants.

Characteristic	Healthy controls	Patients with CKD (n = 25)		Patients on HD (n = 29)		*p* value
		Without DM	With DM	Without DM	With DM	
N	12	14	11	16	13	
Sex (male, %)	100	36	64	25	8	0.001
Age	33.5 ± 13.5	62.5 ± 16.2	64.8 ± 10.4	50.8 ± 14.7	64.6 ± 12.0	< 0.0001
Height (cm)	171.0 ± 4.1	158.3 ± 12.7	159.3 ± 7.3	165.8 ± 5.7	166.8 ± 9.6	0.020
Weight (kg)	63.2 ± 7.8	60.8 ± 15.9	69.9 ± 15.4	59.6 ± 10.4	64.2 ± 13.8	0.325
Body mass index (kg/m^2^)	21.6 ± 2.4	24.0 ± 4.2	27.8 ± 7.2	21.6 ± 3.3	23.0 ± 3.8	0.005
Creatinine (mg/dL)	0.9 ± 0.1	3.3 ± 2.1	2.9 ± 1.3	Renal death (13.0 ± 5.5)	Renal death (8.6 ± 6.8)	< 0.0001
eGFR (mL/min/1.73 m^2^)	81.7 ± 14.6	20.3 ± 16.1	21.0 ± 10.5	Renal death (4.0 ± 1.3)	Renal death (12.9 ± 15.5)	< 0.0001
HbA1c (NGSP) (%)		N/A	6.3 ± 0.9			
Glycoalbumin (%)				N/A	20.0 ± 3.3	
Systolic blood pressure (mmHg)	121.5 ± 8.8	140.9 ± 26.4	129.5 ± 18.5	139.5 ± 30.3	149.8 ± 25.4	0.152
Diastolic blood pressure (mmHg)	81.5 ± 8.0	81.8 ± 18.6	73.8 ± 15.8	82.4 ± 17.5	75.5 ± 12.0	0.531
Hemoglobin (g/dL)	15.2 ± 0.5	10.7 ± 1.6	10.3 ± 2.0	10.2 ± 1.3	10.4 ± 1.8	< 0.0001
Total cholesterol (mg/dL)	189.2 ± 8.8	202.5 ± 56.2	151.8 ± 43.6	151.9 ± 25.2	126.2 ± 19.6	< 0.0001
HDL cholesterol (mg/dL)	54.6 ± 8.4	52.7 ± 25.7	44.3 ± 11.5	48.9 ± 14.0	36.0 ± 8.9	0.068
Coronary artery disease (%)	0	0	9	6	15	0.446
Stroke (%)	0	7	0	6	8	0.803
Smoking habit (%)	8	57	73	38	38	0.020

Values are presented as the mean ± SD or %.

*CKD* chronic kidney disease, *DM* diabetes mellitus, *eGFR* estimated glomerular filtration rate, *HD* hemodialysis, *HDL* high-density lipoprotein, *N/A* not available.

### Bacterial 16S rRNA gene amplicon sequencing and analysis

For 16S rRNA gene sequencing analysis of the human microbiota^18,19^, bacterial genomic DNA was isolated from saliva using an enzymatic lysis method. The isolated DNA (40 ng) was used for PCR amplification of the V1–V2 hypervariable regions of the 16S rRNA gene using the universal primers 27Fmod (5′-AATGATACGGCGACCACCGAGATCTACACxxxxxxxxACACTCTTTCCCTACACGACGCTCTTCCGATCTagrgtttgatymtggctcag-3′) and 338R (5′-CAAGCAGAAGACGGCATACGAGATxxxxxxxxGTGACTGGAGTTCAGACGTGTGCTCTTCCGATCTtgctgcctcccgtaggagt-3′), containing the Illumina Nextera adapter sequence and a unique 8 bp index sequence (indicated by x) for each sample. Amplification was performed on a 9700 PCR system (Life Technologies, Japan) using Ex Taq polymerase (Takara Bio) under the following thermal cycling conditions: initial denaturation at 96 °C for 2 min, 25 cycles of denaturation at 96 °C for 30 s, annealing at 55 °C for 45 s, and extension at 72 °C for 1 min, followed by a final extension at 72 °C. All amplicons were purified using AMPure XP magnetic purification beads (Beckman Coulter) and quantified using the Quant-iT PicoGreen dsDNA assay kit (Life Technologies). Equal amounts of PCR amplicons were mixed and subjected to MiSeq (Illumina) sequencing using the MiSeq reagent kit v2 (500 cycles) according to the manufacturer’s instructions.

After demultiplexing the 16S sequence reads based on the sample-specific index, paired-end reads were joined using the FASTQ-join program. Reads with average quality values < 25 and inexact matches to the universal primer sequences were filtered out; 3000 reads that passed the quality filter were randomly selected from each sample and subjected to downstream analyses. The selected reads were rearranged in descending order according to the quality value^20^ and clustered into operational taxonomic units (OTUs), with a 97% pairwise identity cutoff, using the UCLUST program^21^ version 5.2.32 (http://www.drive5.com/). The taxonomic assignment of each OTU was determined by similarity searching against the Ribosomal Database Project and the National Center of Biotechnology Information genome database using the GLSEARCH program. The sequences determined in the current study were deposited to the DDBJ/GenBank/EMBL databases under the accession number DRA011901.

### Determination of chiral AAs by two-dimensional (2D) high-performance liquid chromatography (HPLC)

D- and L-AAs were evaluated using a 2D HPLC system (Nanospace SI-2 series, Shiseido, Tokyo, Japan), as previously described^22,23^. Briefly, 4-nitrobenzo-2-oxa-1,3-diazole (NBD)-AAs were isolated using an online fraction collecting system in the first dimension with a microbore-ODS column, which was prepared in a fused silica capillary (1000 mm × 0.53 mm i.d., 45 °C; Shiseido). The isolated fractions were automatically transferred to the second dimension, which consisted of a narrow-bore enantio-selective column KSAACSP-001S (250 mm × 1.5 mm i.d, 25 °C; prepared in collaboration with Shiseido), to determine d- and l-enantiomers. The mobile phase for the second dimension was a mixture of methanol and acetonitrile containing formic acid. Fluorescence detection of the NBD-AAs was conducted at 530 nm with an excitation at 470 nm.

### Bacterial culture

Saliva was smeared on sheep blood agar, and incubated at 35 °C for 24 h, after which colonies were isolated, subcultured on sheep blood agar, and incubated at 35 °C for 24 h. Confirmed single colonies were analyzed using matrix-assisted laser desorption/ionization-time of flight mass spectrometry (MALDI/TOF MS; Bruker) and 16S rRNA sequencing to identify the bacterial species. The identified bacteria were cultured in a liquid medium (MH-MP; Nikken Co., Ltd.) for 7 days, centrifuged (3000×*g*, 20 min), the turbidity of the supernatant was adjusted to approximately 2.0 McFarland standard (using a turbidity meter), and chiral AA analysis was performed.

### Statistics

Data, presented as the mean ± standard deviation of the mean, were determined using SPSS Statistics version 23 software (IBM, Tokyo). Statistical analysis was performed using the two-tailed unpaired Student’s *t*-test and Wilcoxon rank-sum test when comparing two groups. One-way ANOVA with Tukey’s multiple comparison test were used for multiple group comparison. Statistical significance was set at *p* < 0.05.

### Ethical approval

This study was approved by the Ethics Committee of the Kanazawa University Hospital (IRB approval No. 1291) and conducted in accordance with the principles of the Declaration of Helsinki. All participants provided written informed consent and were informed of their right to withdraw from the study at any time.

## Results

### D-AA levels in patients with CKD

To investigate the profiles of D-AAs in patients with kidney disease, patients with moderately impaired renal function (patients with CKD) and severely impaired renal function (patients on HD) were included in this study. The clinical characteristics of the patients with kidney disease are summarized in Table 1 and divided into CKD without DM (n = 14), CKD with DM (n = 11), HD without DM (n = 16), and HD with DM (n = 13).

**Figure 1:**
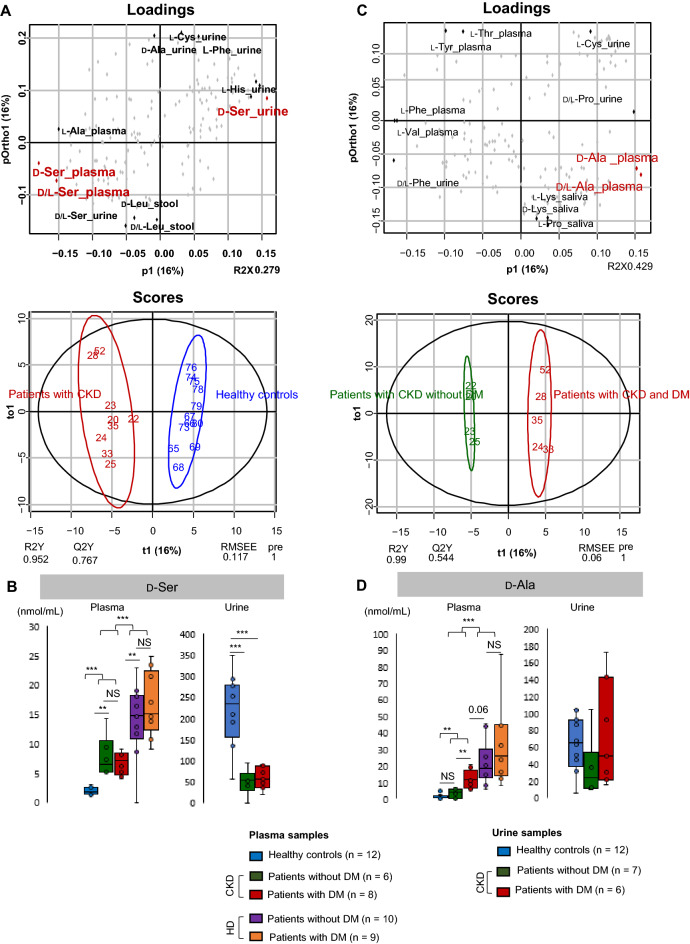
Association between D-serine (D-Ser) and d-alanine (D-Ala) profiles and kidney diseases. (**A**) Orthogonal projection to latent structures discriminant analysis (OPLS-DA) plots for patients with CKD compared with healthy controls. (**B**) Plasma and urine levels of D-Ser in patients with kidney diseases and healthy controls. (**C**) OPLS-DA plots for patients with CKD and DM compared with those without DM. (**D**) Plasma and urine levels of D-Ala in patients with CKD with and without DM and in healthy controls. *p < 0.05, **p < 0.01, ***p < 0.001 (one-way ANOVA). *NS* not significant, *CKD* chronic kidney disease, *DM* diabetes mellitus, *HD* hemodialysis.

Patients with CKD were largely distinguished from healthy controls based on plasma and urine D-Ser levels using orthogonal projection to latent structures discriminant analysis (OPLS-DA) (Fig. 1A). Plasma D-Ser levels were higher (*p* < 0.01), whereas urine D-Ser levels were lower (*p* < 0.001) in patients with CKD than in healthy controls (Fig. 1B). It was suggested that a decrease in urinary D-Ser efflux was associated with an increase in plasma D-Ser in patients with CKD.

Specific D-AAs were evaluated for their ability to identify patients with CKD and DM among those with CKD. Plasma D-Ala levels largely distinguished patients with CKD without DM from those with DM using OPLS-DA (Fig. 1C,D). There was no significant difference in urinary D-Ala levels between healthy controls and patients with CKD.

### D-Ala levels in the stool and saliva

**Figure 2:**
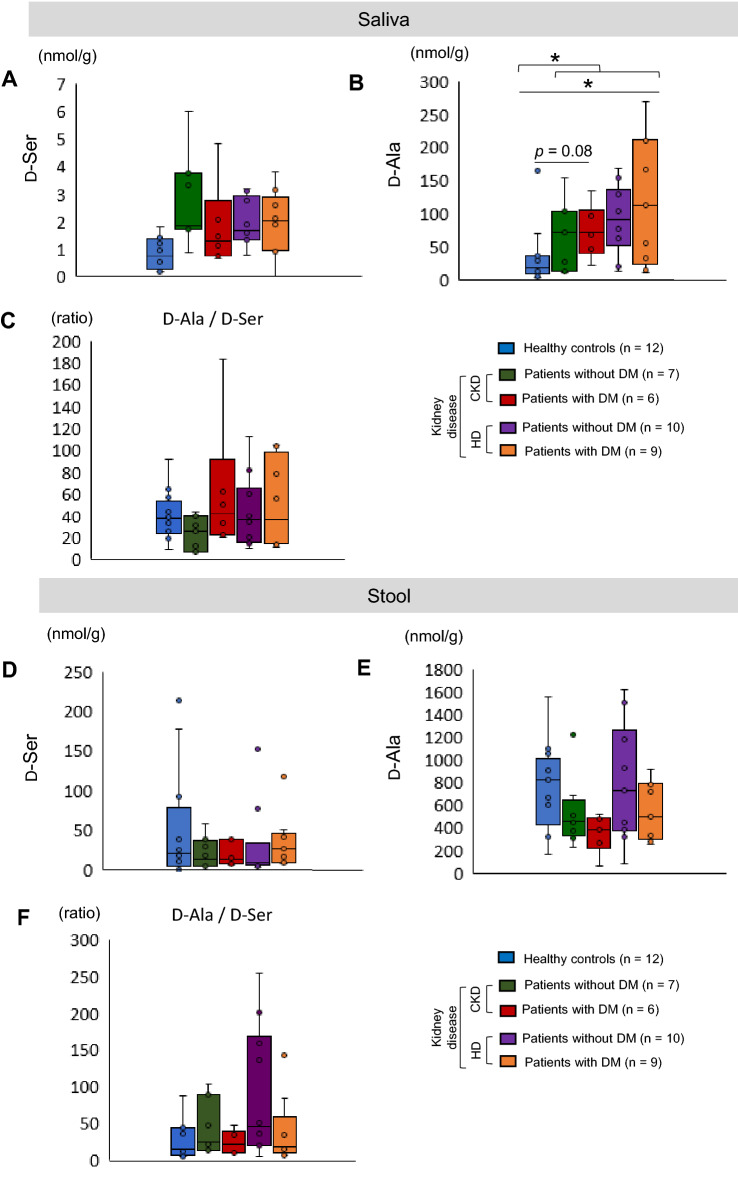
Salivary and stool d-alanine (D-Ala) and D-serine (D-Ser) levels. (**A**) D-Ser and (**B**) D-Ala levels (**C**) D-Ala/D-Ser ratio in saliva samples from healthy controls and patients with kidney diseases. (**D**) D-Ser and (**E**) D-Ala levels (**F**) D-Ala/D-Ser ratio in stool samples from healthy controls and patients with kidney diseases. One-way ANOVA and a *t*-test were used for statistical analysis. *CKD* chronic kidney disease, *DM* diabetes mellitus, *HD* hemodialysis.

We have previously reported that the gut microbiota produces D-Ala in a mouse model of kidney disease^5^. To investigate whether bacteria are the producers of D-Ala in patients with CKD and DM, the oral microbiota (saliva) (Fig. 2A–C) and the gut microbiota (stool) (Fig. 2D–F) were analyzed. The D-Ala levels in the stool were higher than those in the saliva (Fig. 2D,E). Both D-Ser and D-Ala could be detected in the stool and saliva, but the D-Ala levels were higher than those of D-Ser (Fig. 2C,F). The salivary D-Ala levels were higher (*p* < 0.05) in patients with kidney disease than in the healthy controls (Fig. 2B). The stool D-Ala levels did not differ between the healthy controls and patients with kidney disease (Fig. 2E). Based on these results, we speculated that the increased salivary levels of D-Ala were associated with the increased blood D-Ala levels in patients with CKD and DM. Therefore, we focused on the oral microbiota (saliva).

### Changes in the oral microbiota in patients with CKD and DM

**Figure 3:**
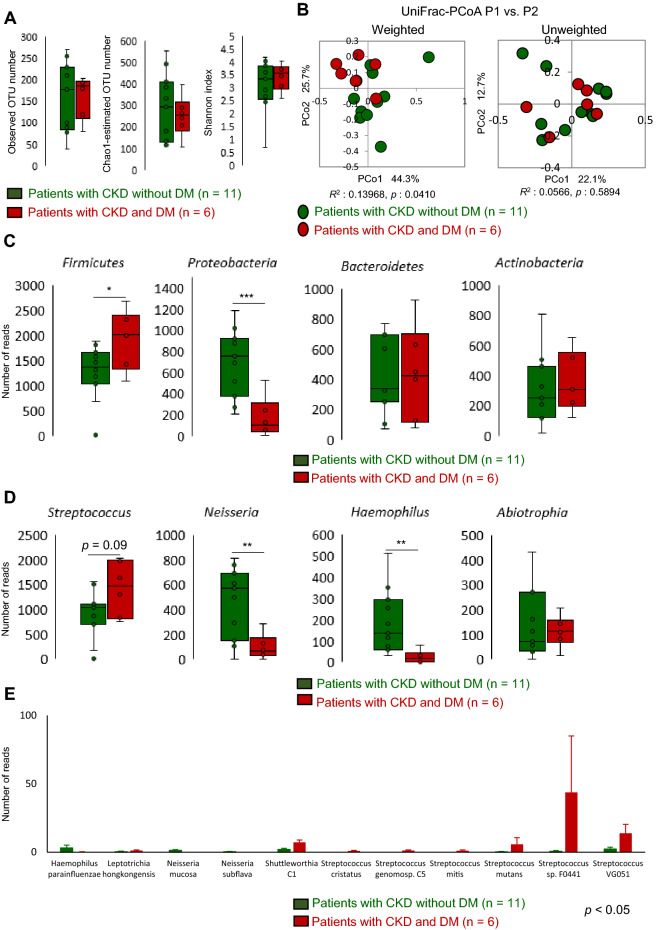
Alpha and beta diversities of the oral microbiota in patients with CKD. (**A**) Differences in alpha diversity between patients with CKD without diabetic kidney disease and those with DM using three indices. (**B**) Differences in beta diversity between patients with CKD with and without DM using weighted and unweighted UniFrac-PCoA. The *R*- and *p*-values obtained using ANOSIM are shown below each graph. (**C**) Phylum, (**D**) genus, and (**E**) species level assignments of the 16S rRNA gene sequence reads between patients with CKD with and without DM. Data in (**A**), (**C**), and (**D**) were statistically analyzed using a *t*-test, and those in (**E**) were analyzed using the Wilcoxon rank-sum test. *p < 0.05, **p < 0.01, ***p < 0.001. *ANOSIM* analysis of similarities, *CKD* chronic kidney disease, *DM* diabetes mellitus, *PCoA* principal coordinates analysis.

We hypothesized that DM conditions in patients with CKD altered the composition of oral bacteria, which in turn contributed to the increased salivary and plasma D-Ala levels in patients with CKD and DM. To identify the bacteria involved, 16S rRNA analysis was performed, to determine the source of the salivary and plasma D-Ala. First, alpha diversity (species richness) of the oral microbiota was analyzed (Fig. 3A, Supplementary Fig. 1A), but no difference was found between patients with CKD without and with DM, as indicated by the observed OTU numbers, Chao1-estimated OTU numbers, and the Shannon index. Further, beta diversity (overall structural similarity and variation) of the oral microbiota was evaluated (Fig. 3B, Supplementary Fig. 1B) using the UniFrac-principal coordinates analysis (PCoA). As expected, patients with CKD and DM formed a unique cluster in weighted UniFrac-PCoA (PERMANOVA *R*^2^ = 0.13968; *p* = 0.041) (Fig. 3B). Patients with DM on HD also formed a unique cluster in unweighted UniFrac-PCoA (PERMANOVA *R*^2^ = 0.14193; *p* = 0.005) (Supplementary Fig. 1B).

**Figure 4:**
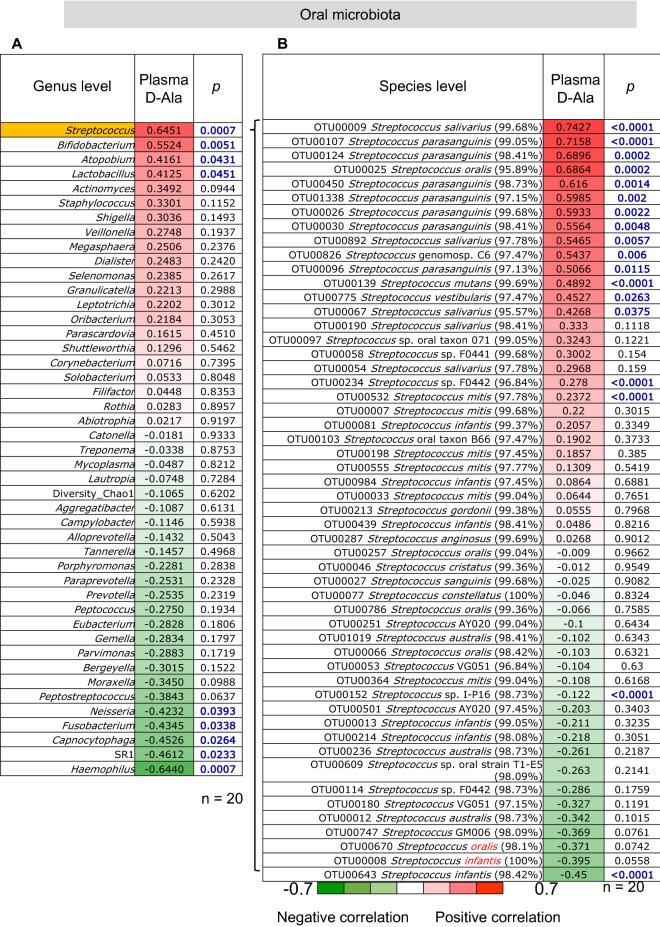
Correlation between the abundance of oral microbiota and plasma d-alanine (D-Ala) levels. (**A**) Correlation between the abundance of oral microbiota at the genus level and plasma D-Ala levels. (**B**) Correlation between the abundance of oral *Streptococcus* species and plasma D-Ala levels.

### Identification of oral bacteria associated with CKD and DM

We attempted to identify the oral bacteria that were particularly unique to patients with CKD and DM. Firmicutes, Proteobacteria, Bacteroidetes, and Actinobacteria were found to be the major phyla in the oral microbiota, with Firmicutes predominating (Supplementary Fig. 2A). A higher abundance of Firmicutes (*p* < 0.05) and a lower abundance of Proteobacteria (*p* < 0.01) were detected in patients with CKD and DM compared with those without DM (Fig. 3C). At the genus level, *Streptococcus* (phylum Firmicutes) comprised the majority of bacteria in both the healthy controls and patients (Supplementary Fig. 2B). The abundance of *Streptococcus* tended to be higher (*p* = 0.09) in patients with CKD and DM than in those without DM (Fig. 3D). The abundances of *Neisseria* and *Haemophilus* (phylum Proteobacteria) were lower (*p* < 0.01) in patients with CKD and DM than in those without DM (Fig. 3D). The abundance of some *Streptococcus* species was higher (*p* < 0.05) in patients with CKD and DM than in those without DM (Fig. 3E). In addition, in the group of patients who were on HD, the abundance of *Streptococcus* species was higher (*p* < 0.05) in patients with DM than in those without DM (Supplementary Fig. 1E).

### Correlation between the abundance of oral *Streptococcus* and D-Ala levels

We investigated the correlation between the abundance of oral *Streptococcus* and plasma and salivary levels of D-Ala. At the genus level, the abundance of *Streptococcus* showed a significant positive correlation (*r* = 0.65, *p* < 0.001) with plasma D-Ala levels (Fig. 4A) and a positive correlation (*r* = 0.16) with salivary D-Ala levels (Supplementary Fig. 3). At the species level, the abundances of *Streptococcus* species such as *S. salivarius, S. parasanguinis*, and *S. oralis*, were significantly positively correlated (*p* < 0.05) with plasma D-Ala levels (Fig. 4B).

### D-Ala production by oral *Streptococcus* spp.

**Figure 5:**
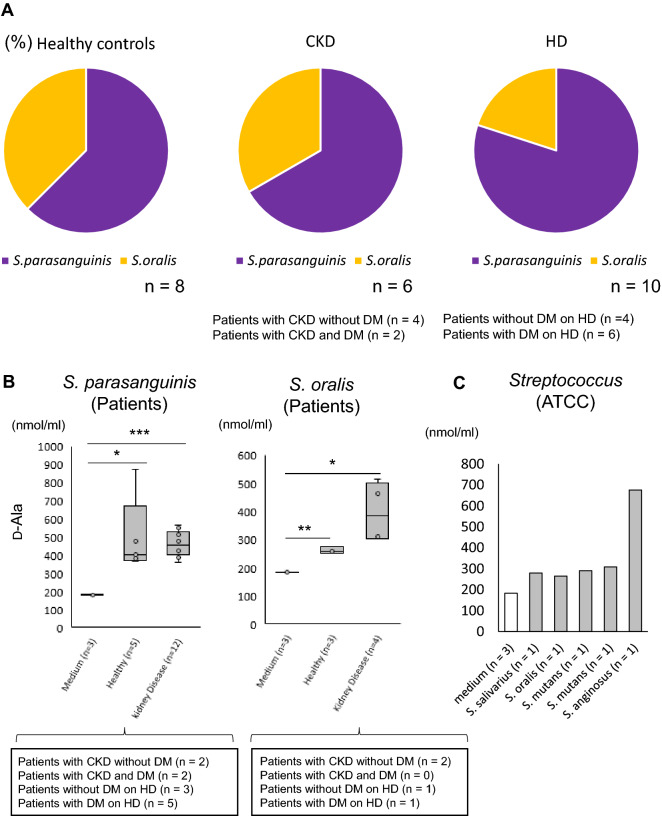
Association between oral *Streptococcus* species and d-alanine (D-Ala) levels. (**A**) Rates of isolation of *Streptococcus* species from saliva. (**B**) D-Ala levels in the culture supernatants of *S. parasanguinis* and *S. oralis*. (**C**) D-Ala levels in the culture supernatants of *Streptococcus* species from the American Type Culture Collection. *p < 0.05, **p < 0.01, ***p < 0.001 (one-way ANOVA). *CKD* chronic kidney disease, *DM* diabetes mellitus, *HD* hemodialysis.

To examine whether increases in the abundance of oral *S. salivarius, S. parasanguinis*, and *S. oralis* were associated with D-Ala production, we isolated *Streptococcus* species from saliva samples. Twenty-four strains of *Streptococcus* were isolated from healthy controls (n = 8) and patients with kidney disease (n = 16) (Fig. 5). These *Streptococcus* strains were identified as *S. parasanguinis* and *S. oralis* using MALDI/TOF MS (Supplementary Table 1). The rates of isolation from healthy controls, patients with CKD, and patients with CKD and HD are shown in Fig. 5A. D-Ala levels in the culture supernatants of *S. parasanguinis* and *S. oralis* were higher than those in the medium (Fig. 5B). The amounts of D-Ala produced were similar between the strains from the healthy controls and patients with kidney disease. *Streptococcus* species that could not be isolated were purchased from the American Type Culture Collection (ATCC). The D-Ala levels in the culture supernatants of the *Streptococcus* species from ATCC tended to be higher than those in the medium (Fig. 5C).

## Discussion

**Figure 6:**
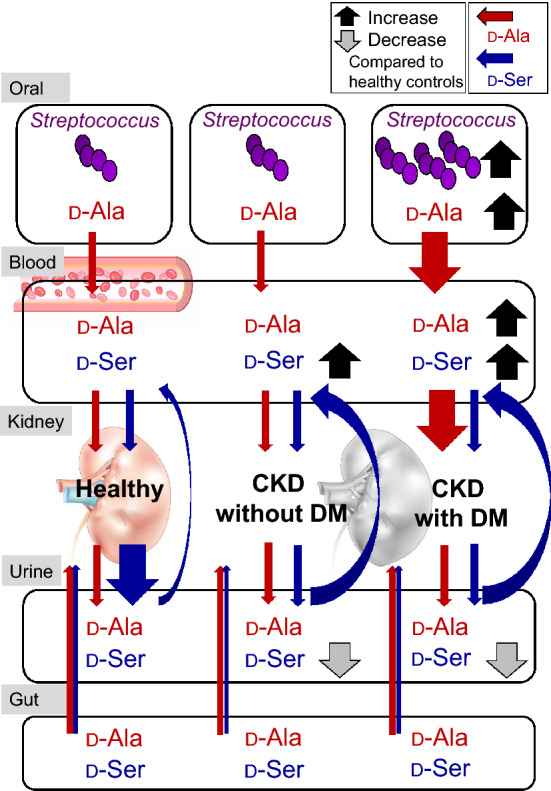
Proposed model of the relationship between oral *Streptococcus*-derived d-alanine (D-Ala) and kidney disease. d-Serine (D-Ser) and D-Ala were present in the blood and urine of healthy controls. Plasma D-Ser levels were higher, whereas urine D-Ser levels were lower in all patients with CKD than in healthy controls. However, the abundances of oral *Streptococcus* species and plasma D-Ala levels were higher in patients with CKD and DM than in those without DM. In addition, salivary D/L-Ala and plasma D-Ala levels were associated with positive estimated glomerular filtration rate slopes in patients with CKD and DM. *CKD* chronic kidney disease, *DM* diabetes mellitus.

In this study, we found that patients with CKD and DM have a unique oral microbiota and chiral amino acid profile. Oral *Streptococcus* produced D-Ala. This D-Ala was increased in the blood and may be a useful biomarker for patients with CKD and DM (Fig. 6).

D-Ala levels in the blood were higher in patients with CKD and DM than in patients with CKD. Urinary D-Ser levels were decreased in patients with CKD and DM compared to healthy subjects, suggesting that the increase in blood D-Ser levels were partly due to the decreased urinary excretion. Interestingly, urinary D-Ala levels did not change between healthy subjects and patients with CKD and DM. Thus, we sought to identify the major sources of D-Ala production to elucidate the mechanism by which blood D-Ala is increased in patients with CKD and DM. The main sources of D-Ala are believed to be the microbiota, diet, and endogenous biosynthesis. We have previously found that many D-AAs, including D-Ser and D-Ala, are not detectable in stools of germ-free mice but are observed in those of normal mice^10,11^. In addition, many bacterial species encode alanine racemase, which is a D-Ala synthase^24,25^; D-Ala is required for the peptidoglycan component in the bacterial cell wall. These suggest bacterial production of D-Ala.

We focused on the microbiota to investigate the mechanism of the increase of D-Ala levels in plasma. Saliva and stool were collected from patients with CKD as samples of the oral and gut microbiota, respectively, and both microbiotas were found to produce D-Ala. Salivary levels of D-Ala were higher in patients with CKD than in healthy controls. We have previously confirmed in an AKI mouse model that D-Ala was transferred into the blood by drinking water administration and was renoprotective^11^. Therefore, plasma D-Ala levels may have increased in patients with CKD owing to an increase in salivary D-Ala levels.

In elucidating the mechanism underlying the increase in salivary D-Ala levels compared with those in healthy controls, we found that the oral microbiota was altered. Some reports have suggested an association between oral bacteria and DM^26,27^, CKD^28^, and metabolic syndrome^29^. In the present study, we identified changes in the oral microbiota profiles in patients with CKD and DM. The number of oral *Streptococcus* species was higher in patients with CKD and DM than in those without DM. Given that D-Ala production by *Streptococcus* species is similar in patients with kidney disease and healthy controls, the increased levels of salivary and plasma D-Ala may be associated with the increased number of *Streptococcus* species in saliva from patients with CKD and DM.

The D-Ala levels in the body are also affected by other factors, such as the diet and circadian rhythm. Some food types, particularly fermented foods, are rich in D-AAs and affect D-Ala levels in the body^30,31^. In addition, circadian rhythm-related fluctuations in D-Ala levels have been observed in the pituitary gland, pancreas, blood, and urine^32,33^. For maximum elimination of these effects, samples in this study were taken at fasting levels early in the morning. Some studies have reported that D-Ala levels were increased by endogenous synthesis in local tissues. High D-Ala levels have been detected in the rat pancreas (29.2 ± 5.0 nmol/g) and anterior pituitary gland (86.4 ± 9.9 nmol/g)^34^. Therefore, in addition to the microbiota, the association of D-Ala levels with the anterior pituitary gland and pancreas should be investigated in the future. Moreover, the effects of age, gender, dietary habits, and medications on D-Ala levels were not clear. Therefore, the mechanisms of regulation of D-Ala levels in the body requires further investigation.

The present study has several limitations. There were considerable differences in age and sex between healthy controls and patients with CKD. Additionally, this was a single-center study with a small sample size. Therefore, larger multicenter studies should be conducted to confirm our findings. Although patients taking antibiotics were excluded, other medications may have affected the abundance of the microbiota.

Collectively, the present study revealed higher plasma D-Ser concentrations in CKD patients and higher plasma D-Ala concentrations in CKD and DM patients. It was suggested that oral *Streptococcus* was involved in the cause of increased plasma D-Ala in CKD and DM patients. These findings introduce a potentially new concept in nephrology and may contribute to the diagnosis of patients with CKD and DM.

## Supplementary Information

Supplementary Information.

## Data Availability

The 16S rRNA gene V1–V2 region sequences analyzed in the current study have been deposited to the DDBJ/GenBank/EMBL databases under accession number DRA011901.
