# Supplementary material for: Increased levels of oral Streptococcus-derived d-alanine in patients with chronic kidney disease and diabetes mellitus
Source: Sci Rep. 2022 Dec 16;12:21773. doi: 10.1038/s41598-022-26175-1 (PMC9758232; doi:10.1038/s41598-022-26175-1)
Supplement: Supplementary file 1 — Supplementary Information. [file 41598_2022_26175_MOESM1_ESM.docx]

**Supplemental Material**


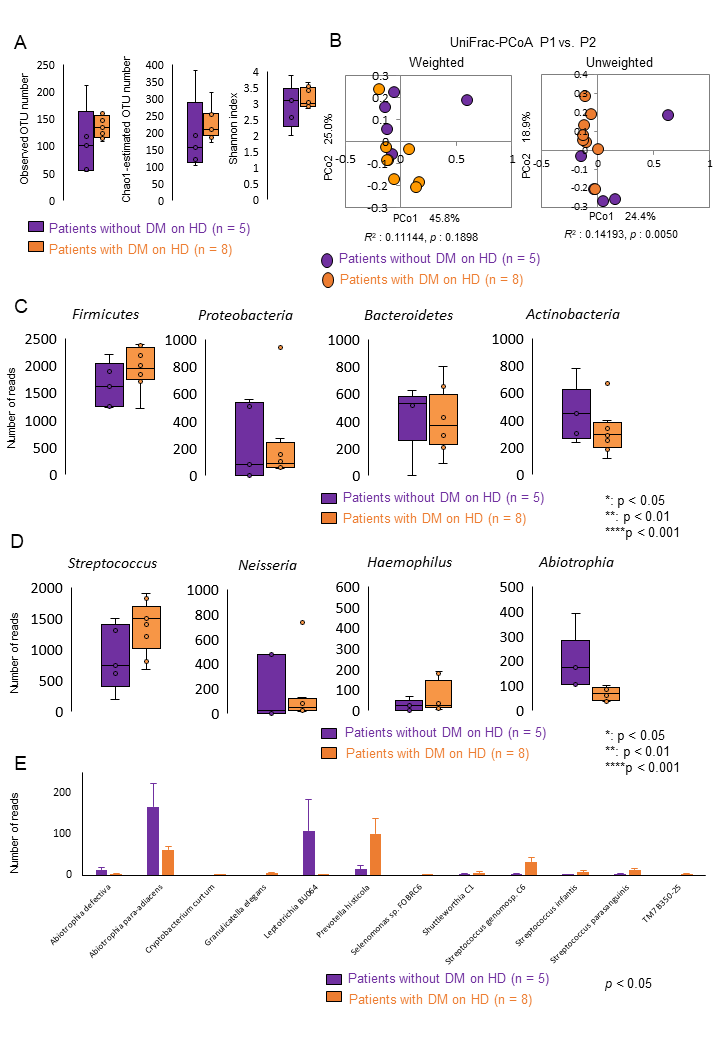
Supplemental Figure 1. Alpha and beta diversities of the oral microbiota in patients with and without DM on HD. (A) Differences in alpha diversity between the two groups are shown by three indices. (B) Differences in beta diversity between the two groups are based on weighted and unweighted UniFrac-PCoA. Analysis of similarities *R*- and *p*-values between the two groups are shown below each graph. (C) Phylum, (D) genus, and (E) species level assignments of 16S rRNA gene sequence reads in the two groups. Data in (A), (C), and (D) were statistically analyzed using a *t*-test, and those in (E) were analyzed using the Wilcoxon rank-sum test. DKD, diabetic kidney disease; HD, hemodialysis; PCoA, principal coordinates analysis.


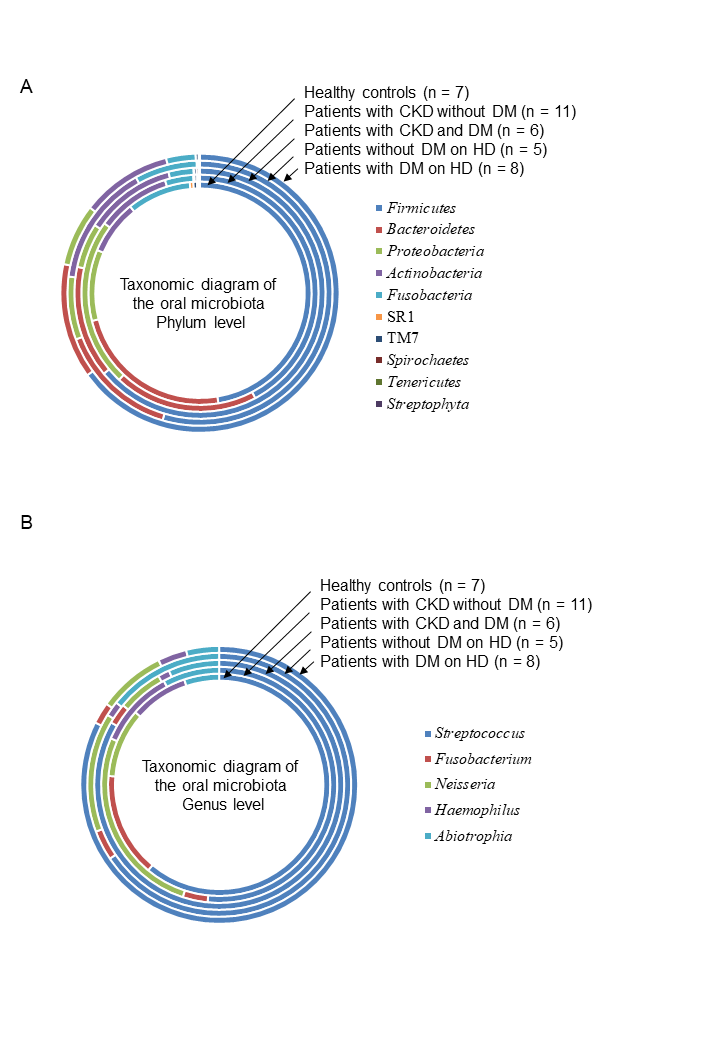
Supplemental Figure 2. Taxonomic diagrams of the oral microbiota from patients with kidney disease at the (A) phylum and (B) genus levels.


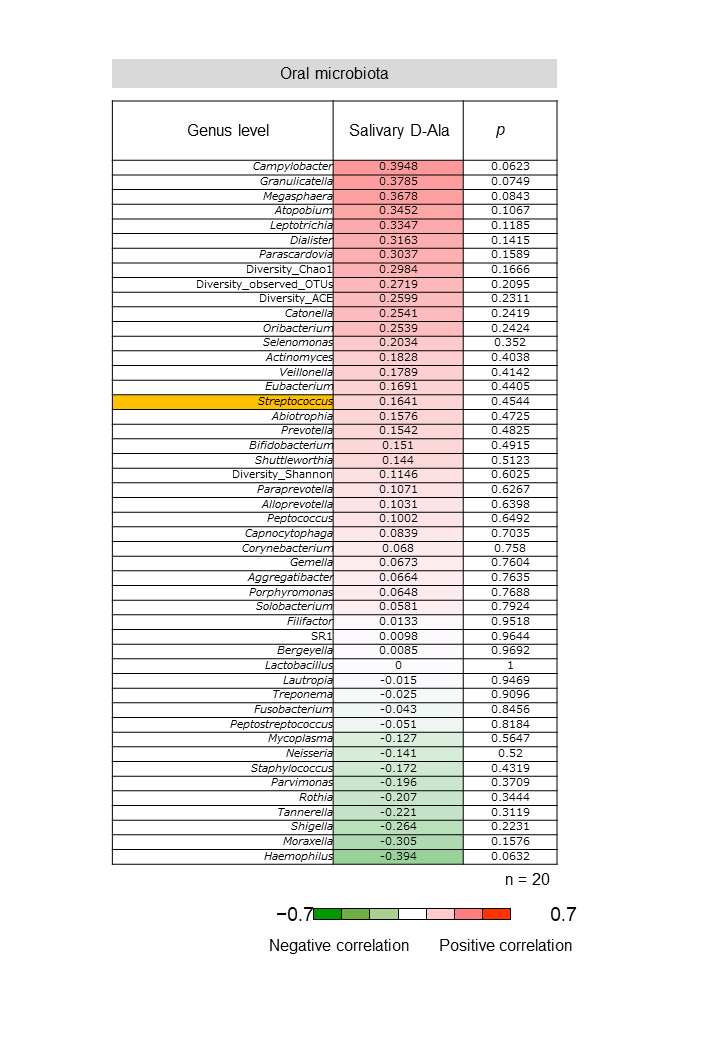
Supplemental Figure 3. Correlation between the abundances of oral microbiota genera and salivary D-Ala levels.

Supplemental Table 1. Matrix-assisted laser desorption/ionization–time of flight mass spectrometry (MALDI/TOF MS) scores of *Streptococcus* isolates from study subjects.

| **Subject** | ***Streptococcus* Species** | **MALDI/TOF MS Score** |
| --- | --- | --- |
| Healthy control | *S. parasanguinis* | 1.7 |
| Healthy control | *S. parasanguinis* | 1.99 |
| Healthy control | *S. parasanguinis* | 1.84 |
| Healthy control | *S. parasanguinis* | 2.21 |
| Healthy control | *S. oralis* | 1.76 |
| Healthy control | *S. oralis* | 1.83 |
| Healthy control | *S. parasanguinis* | 2 |
| Healthy control | *S. oralis* | 1.94 |
| Patient with CKD without DM | *S. oralis* | 2.23 |
| Patient with CKD without DM | *S. parasanguinis* | 1.8 |
| Patient with CKD without DM | *S. parasanguinis* | 1.76 |
| Patient with CKD without DM | *S. oralis* | 2.14 |
| Patient with CKD and DM | *S. parasanguinis* | 2.25 |
| Patient with CKD and DM | *S. parasanguinis* | 2.11 |
| Patient without DM on HD | *S. parasanguinis* | 2.03 |
| Patient without DM on HD | *S. oralis* | 1.74 |
| Patient without DM on HD | *S. parasanguinis* | 2.13 |
| Patient without DM on HD | *S. parasanguinis* | 1.86 |
| Patient with DM on HD | *S. parasanguinis* | 1.76 |
| Patient with DM on HD | *S. parasanguinis* | 2.09 |
| Patient with DM on HD | *S. oralis* | 1.95 |
| Patient with DM on HD | *S. parasanguinis* | 2.22 |
| Patient with DM on HD | *S. parasanguinis* | 2.09 |
| Patient with DM on HD | *S. parasanguinis* | 2.16 |

Score: 2.000–2.299, secure genus identification and probable species identification; 1.700–1.999, probable species identification.
